# Physiotherapy after tibial plateau fracture fixation: A systematic review of the literature

**DOI:** 10.1177/2050312120965316

**Published:** 2020-10-14

**Authors:** Efthymios Iliopoulos, Nikiforos Galanis

**Affiliations:** 1 Brighton & Sussex University Hospitals, Brighton, UK; 2 Division of Sports Medicine, Department of Orthopaedics, Papageorgiou General Hospital, Medical School, Aristotle University of Thessaloniki, Thessaloniki, Greece

**Keywords:** Physical therapy, tibia plateau, proximal tibia, rehabilitation

## Abstract

**Background:**

Tibial plateau fractures are frequent injuries that orthopaedic surgeons face. It has been reported that they have a significant negative impact on the patients’ lives, decreasing their quality of live, keeping them of work for long periods of time and reducing their activity levels.

**Aim:**

Interestingly, there is not enough focus in the literature about the post-operative rehabilitation of these patients. The aim of the present review is to investigate this field of the literature and try to give answers in four main questions: the range of motion exercises post-surgery, the immobilisation, the weight-bearing status and the ongoing rehabilitation.

**Materials and Methods:**

A literature search was conducted using the PubMed and the Google Scholar search engines. A total of 39 articles met the criteria to be included in the study.

**Results:**

The literature about this subject is scarce and controversial. Early range of motion exercises should be encouraged as soon as possible after the procedure. The immobilisation after plate fixation does not seem to be correlated with any benefits to the patients. The weight-bearing status of the patients was the most controversial in the literature with the early weight-bearing gaining ground at the most recent studies. Tibia plateau fractures can have significant impact on the patients’ lives, so ongoing rehabilitation with focus on quadriceps strengthening and proprioception exercises is recommended.

**Conclusion:**

The present literature review illuminates the controversy that exists in the literature about the physiotherapy following tibia plateau fracture fixation. Early range of motion exercises and early weight bearing should be encouraged. Immobilisation does not seem to provide any benefit. Ongoing rehabilitation should be considered with the view of better clinical outcomes.

## Introduction

Intra-articular fractures of the proximal tibia are commonly referred as tibial plateau fractures. They are quite common injuries and represent about 1% of all fractures in adult population. The mean age of the patients sustaining these types of fractures is approximately 52 years.^1^ There are two main age groups of these patients. The first one is young male patients who sustain injuries after high-energy trauma (road traffic accidents) and the second is older female patients who sustain these injuries after low-energy trauma (simple falls). The high-energy trauma leading to increased axial and/or torsional forces to the proximal tibia is the main causative factor for the first group of patients. The fragility of the bone due to osteoporosis, despite the low forces sustained during trauma, is the main causative factor for the older group of patients.^2,3^

It is well documented in the literature that these types of fractures have a significant impact on the patients’ lives and the health care systems. It is reported that these patients are not able to return to work for 3–4 months after surgical fixation.^4^ Multiple surgical complications such as wound complications, infection, bleeding and metalwork problems can increase the burden of poor outcomes.^5,6^ Other factors related to the injury itself, such as later development of arthritis, muscle and bone atrophy, and joint stiffness, can affect the patients’ lives significantly leading to ongoing functional problems and increased socio-economic burden.^4,6^ Physical therapy is a very important part of the patients’ rehabilitation during their journey to return to their pre-injury activity levels or as close as they can to that state. It can help prevent some of these problems or focus in the areas that these patients need, to achieve better outcomes.

Surprisingly, in the literature, there is not a lot of information about the rehabilitation of these patients. Most of the research articles focus on either the type of fixation of the tibial plateau fractures or the clinical outcomes after fixation. This study focuses on the physiotherapy that these patients should receive. The aim of this study is to review the current literature about the rehabilitation of patients who sustained a tibial plateau fracture and treated surgically, with the view to provide some guidance especially on four main subjects: the range of motion exercises of the knee joint, immobilisation, weight bearing and ongoing rehabilitation.

## Materials and methods

A systematic review of the current literature was performed using the search engines MEDLINE and Google Scholar. The keywords used were as follows: ‘tibia plateau’ OR ‘tibial plateau’ AND ‘physiotherapy’ OR ‘rehabilitation’ OR ‘physical therapy’. Inclusion criteria were articles written in English which contain information about the rehabilitation of patients who surgically fixed for tibia plateau fractures. Articles not written in English, studies performed on non-human subjects or articles not mentioning the rehabilitation protocol of the patients were excluded from the study.

Two blinded examiners (E.I., N.G.) performed the search in the above search engines using the above-mentioned search criteria. They independently examined the articles yielded by the search in terms of the inclusion/exclusion criteria. The final number of articles extracted was correlated between the two examiners. Duplicates were removed, and for the final number of articles included in the study, the full texts were obtained for the final data extraction. The two examiners then independently extracted the relevant data from the included papers. Then, the results were combined for each research question forming the final draft of the study.

## Results

The above-mentioned search methodology yielded 56,835 articles. After removing the duplicates, non-relevant articles and articles fitting the above-mentioned exclusion criteria, a total of 39 articles were included in this study.

**Figure 1.**
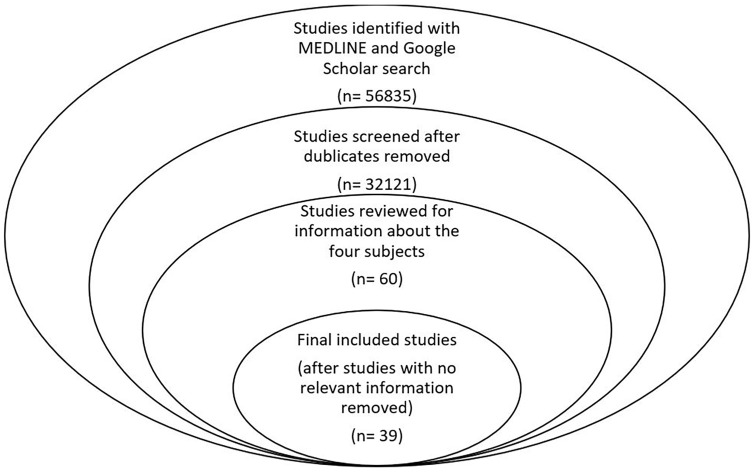
PRISMA flow diagram.

From the articles included in the study, information was extracted about the four subjects including the range of motion of the knee joint, the immobilisation, the weight-bearing status and the ongoing rehabilitation of the patients after fixation of tibia plateau fractures. All the information extracted is presented separately for each subject in the discussion. Figure 1 demonstrates the PRISMA (Preferred Reporting Items for Systematic Reviews and Meta-Analyses) flow diagram of the study.

## Discussion

To the authors’ surprise, the amount of the relevant information about the rehabilitation of patients undergoing surgical fixation for their tibial plateau fractures is very scarce in the literature and sometimes significantly diverse. In the following paragraphs, the authors try to present all the available information so far about each subject. As the rehabilitation protocols can be very different between internal fixation and fixation with circular frame, the discussion is presented with clear distinction between the two surgical techniques.

### Range of motion exercises

The importance of the early mobilisation and range of motion exercises of the knee joint has been obvious in the literature for over 40 years,^7^ but there is a variety of options regarding the timing and the type of the range of motion exercises after internal fixation of tibial plateau fractures. These could be passive, active or active-assisted exercises and can start immediately after the operation at the second post-operative day or later once the wounds heal.^6,8,9^ Sinha et al.^10^ in their study allowed the patients to perform range of motion exercises early driven by the success of the post-operative pain control (5–14 days after the operation). Closed-chain kinetic exercises and AC sine-wave stimulation and laser therapy have been used as well from other authors.^11^ Blokker et al.^12^ did not find any benefit in the long-term outcomes, on starting the range of motion exercises of the knee joint immediately after the operation or 2 weeks post-operatively.

The continuous passive motion (CPM) devices are frequently used to aim the range of motion of these patients after surgical fixation.^13^ Nearly half of the studies were found to use these devices in a recent scoping review by Arnold et al.,^14^ but it is still unclear in the literature about the exact amount of influence on patient outcomes and prevention of complications.^15^

The range of motion exercise protocols can be altered from the surgical technique used by the surgeons. When a circular frame is used and the knee joint is bridged with the frame, the start of the range of motion exercises of the knee is delayed until after the distal femoral ring is removed (usually 6–8 weeks after the application of the frame).^16^ On the contrary, if the knee is not bridged with the circular frame, the range of motion exercises of the knee joint is encouraged as early as possible.^13,17,18^

### Immobilisation

The immobilisation after the fixation of the tibial plateau fractures applies only to the internal fixation surgical option and includes the use of a hinged knee brace. The use of the brace could vary from 10 days to 6 weeks.^19,20^ The use of braces as a post-operative type of immobilisation is not frequently reported in the literature, with only one-third of the studies found by Arnold et al.^14^ recommending its use.

Chauhan et al. performed the first prospective randomised trial about the use of bracing after internal fixation for tibial plateau fractures. Even though the study was underpowered, their results showed no differences in clinical, subjective reported and radiological outcomes.^21^ On the contrary, Polat et al.^22^ in their case series found that the patients who were immobilised for more that 6 weeks post-operatively had poorer functional outcomes at their final follow-up, so they proposed not to immobilise the patients more than 2 weeks post-operatively.

### Weight bearing

The literature about the weight-bearing status of the patients post-operatively following internal fixation is more diverse compared to the literature about the circular frame fixation which is more homogeneous leaning more towards full weight bearing.

The weight-bearing status of the patients after internal fixation has been the most controversial issue in the literature. Van der Vusse et al.^23^ illuminated this issue very well with their study, reporting a variety of compliance with AO guidelines among surgeons, about the weight-bearing status post-operatively. This can vary from immediate weight bearing as pain allows after internal fixation, to partial weight bearing for 6–12 weeks and non-weight bearing from 4 to 12 weeks. These states progress gradually to full weight bearing after the above-mentioned periods of time.^2,9–11,19,24–27^ Other authors use the radiographic union as a marker to progress to full weight bearing.^6,10,18,28–30^ Most frequently, a variety of partial weight-bearing protocols for 4–6 weeks is preferred by the surgeons.^14^ Interestingly, Thewlis et al.^31^ have shown with their gait analysis study that patients who are instructed to partial weight bear, they are self-regulating their weight-bearing status, but nevertheless this fact did not affect the outcomes. Kalmet et al. in their retrospective study comparing partial weight bearing with restrictive weight bearing after plate fixation of tibial plateau fractures report no differences in terms of complications and patient reported outcome measure (PROM) outcomes. They found that the patients in the partial weight-bearing group were able to achieve full weight bearing significantly earlier.^32^ Polat et al.^22^ in their study found that if the full weight bearing was delayed more than 12 weeks the functional outcomes were poorer, thus they recommend partial weight bearing for 6 weeks followed by full weight bearing between 6 and 12 weeks post-operatively.

Traditionally, early weight bearing of the patients after internal fixation for the tibia plateau fractures was linked with the fear of loss of reduction, metalwork failure or malunion. Nevertheless, single cohort studies by Thewlis et al.^31^ and Solomon et al.^33^ showed that immediate weight bearing after plate fixation did not increase the incidence of loss of reduction or metalwork failure and does not affect the clinical outcomes at 1 year post-operatively. Thewlis et al.^27^ had in a previous study shown that there is no correlation between the peak loading during walking at self-selected speeds and fracture migration, explaining that the forces during gait are not enough to exceed the elastic limit of the bone/fixation construct. It is reported that the energy expenditure burden of non and partial weight bearing is substantial.^8^ Haak et al.’s study^34^ and more recently Williamson et al.’s^35^ study compared two cohorts of patients treated with plate fixation for tibial plateau fractures, finding no differences between the two cohorts in terms loss of reduction or metalwork failure. In addition, Carrera et al.^36^ in their biomechanical study demonstrated that the use of locking plate for spit-type tibia plateau fractures provides a more stable construct than screw fixation, proposing that immediate full weight bearing can be commenced for these types of fixations. In Polat et al.’s^22^ study, the differences in outcomes between plate and screw fixation were not significant.

However, the literature about the weight-bearing status after circular frame fixation is more homogeneous with early weight bearing being the prevalent option of the surgeons.^13,37^ Elsoe et al.^17^ kept their patients treated with circular frame non-weight bearing for the first 6 post-operative weeks.

### Ongoing rehabilitation

Fractures of the tibia plateau had a significant impact on the patients’ lives, decreasing their quality of life and reducing the involvement with sporting activities. Robertson et al.^38^ in their review study report that patients sustaining a tibia plateau fracture have lower return to sport rates compared to other injury types. Kraus et al.^39^ found significant decline of these patients’ sporting activity levels 2 years post treatment. Quintens et al. demonstrated in their study that especially tibia plateau fractures which involve the posterior column lead to reduced engagement with sports and patients’ dissatisfaction. Less than 1 in 5 patients return to pre-injury levels of activity with reported main issues such as pain, knee joint stiffness, instability and fear for re-injury.^40^ This fact illuminates the need for ongoing rehabilitation of these patients, in order to try to improve the patients’ satisfaction and level of activity.

In the literature, there are three studies investigating the gait pattern of patients who sustained a tibia plateau fracture. In the short term after the frame removal, the patients return to normal morphology of gait with some impairments especially during the single leg support and the terminal stance phase of the gait circle.^41^ Elsoe and Larsen^42^ report increased asymmetry of the gait pattern of these patients 1 year after the frame removal. There was a moderate association between this asymmetry and the patients’ health-related quality of life. Similar were the results of Warschawski et al.^43^ who found significant correlation between SF-12 scores and gait impairments even 3 years after injury. These findings illuminate the need for early start but ongoing rehabilitation of these patients with increased focus on the quadriceps strengthening and proprioception exercises with the view to avoid these impairments and improve the long-term outcomes.^41^ Sinha et al.^10^ in their recent study started isokinetic exercises of the quadriceps as early as the second post-operative day.

### Limitations and future studies

To the authors’ knowledge, this review is the first in the literature which tries to illuminate the tendencies of the present literature about the physiotherapy strategies following the fixation of tibia plateau fractures. Unfortunately, the literature at the moment is not very rich in this subject, with a severe lack of comparative and randomised controlled studies. Most of the extracted information is information extracted from studies which had different aims, other than the rehabilitation of the patients. Nevertheless, this review is a start on pointing the literature in this interesting direction as well.

Future studies that compare different physiotherapy post-operative protocols would be interesting and will point towards the correct direction the orthopaedic surgeons. Falzarano et al. in their study about the gait pattern after distal tibia pilon type fractures found that the results of different fixation types are similar as long as the fracture reduction and limb alignment was restored. Expanding this concept to the proximal tibia and tibia plateau fractures would be interesting, investigating if there are any differences between the types of fixation and gait pattern outcomes for the patients with tibia plateau fractures.^44^

## Conclusion

Despite the increased frequency of the tibial plateau fractures and the significant impact they have on the patients’ lives, the current literature remains scarce and controversial about the physiotherapy that these patients should receive. The last few years there is a tendency for this problem to be solved by some recently published papers exploring this field. At the moment, the most consensus lies about the importance of early range of motion exercise of the knee joint. The use of CPM device has not been linked with better outcomes. Brace immobilisation does not seem to provide any advantages. The most controversy exists on the post-operative weight-bearing status of these patients, with more aggressive approaches gaining ground the last years. About the ongoing rehabilitation the literature is very poor, but an increased focus on continuous quadriceps strengthening should be considered.
